# Bioinformatic workflows for deriving transcriptomic points of departure: current status, data gaps, and research priorities

**DOI:** 10.1093/toxsci/kfae145

**Published:** 2024-11-05

**Authors:** Jason O’Brien, Constance Mitchell, Scott Auerbach, Liam Doonan, Jessica Ewald, Logan Everett, Adam Faranda, Kamin Johnson, Anthony Reardon, John Rooney, Kan Shao, Robert Stainforth, Matthew Wheeler, Deidre Dalmas Wilk, Andrew Williams, Carole Yauk, Eduardo Costa

**Affiliations:** Ecotoxicology and Wildlife Health Division, Environment and Climate Change Canada, Ottawa, ON J8X 4C6, Canada; Health and Environmental Sciences Institute, Washington, DC 22205, United States; Predictive Toxicology Branch, Division of Translational Toxicology, NIEHS, Research Triangle Park, NC 27709, United States; Syngenta International Research Centre, Berkshire RG42 6EY, United Kingdom; Institute of Parasitology, McGill University, Montreal, QC H3A 0G4, Canada; Center for Computational Toxicology and Exposure, Office of Research and Development, US Environmental Protection Agency, Research Triangle Park, NC 27709, United States; FMC Agricultural Solutions, Newark, DE 19711, United States; Corteva Agriscience, Indianapolis, IN 46268, United States; Healthy Environments and Consumer Safety Branch, Health Canada, Ottawa, ON K1A 0K9, Canada; Existing Substances Risk Assessment Bureau, Health Canada, Ottawa, ON K1A 0K9, Canada; Syngenta Crop Protection, LLC, Greensboro, NC 27409, United States; Department of Environmental and Occupational Health, School of Public Health—Bloomington, Indiana University, Bloomington, IN 47405, United States; Radiation Protection Bureau, Health Canada, Ottawa, ON K1A 0K9, Canada; Predictive Toxicology Branch, Division of Translational Toxicology, NIEHS, Research Triangle Park, NC 27709, United States; Investigative Safety, GSK, Collegeville, PA 19426, United States; Environmental Health Science and Research Bureau, Health Canada, Ottawa, ON K1A 0K9, Canada; Department of Biology, University of Ottawa, Ottawa, ON K1N 6N5, Canada; Corteva Agriscience, 71 Mogi Mirim, Brazil

**Keywords:** transcriptomics, POD, bioinformatics

## Abstract

There is a pressing need to increase the efficiency and reliability of toxicological safety assessment for protecting human health and the environment. Although conventional toxicology tests rely on measuring apical changes in vertebrate models, there is increasing interest in the use of molecular information from animal and in vitro studies to inform safety assessment. One promising and pragmatic application of molecular information involves the derivation of transcriptomic points of departure (tPODs). Transcriptomic analyses provide a snapshot of global molecular changes that reflect cellular responses to stressors and progression toward disease. A tPOD identifies the dose level below which a concerted change in gene expression is not expected in a biological system in response to a chemical. A common approach to derive such a tPOD consists of modeling the dose–response behavior for each gene independently and then aggregating the gene-level data into a single tPOD. Although different implementations of this approach are possible, as discussed in this manuscript, research strongly supports the overall idea that reference doses produced using tPODs are health protective. An advantage of this approach is that tPODs can be generated in shorter term studies (e.g. days) compared with apical endpoints from conventional tests (e.g. 90-d subchronic rodent tests). Moreover, research strongly supports the idea that reference doses produced using tPODs are health protective. Given the potential application of tPODs in regulatory toxicology testing, rigorous and reproducible wet and dry laboratory methodologies for their derivation are required. This review summarizes the current state of the science regarding the study design and bioinformatics workflows for tPOD derivation. We identify standards of practice and sources of variability in tPOD generation, data gaps, and areas of uncertainty. We provide recommendations for research to address barriers and promote adoption in regulatory decision making.

Traditionally in toxicology, whole animal studies are designed to identify a dose level at which no adverse effects are likely to occur for humans or species of concerns. These reference doses are extrapolated from no adverse effect levels or benchmark doses (BMDs) that are used as a point of departure (POD). Transcriptomic dose–response modeling is a promising method that can produce a POD that has potential applications in quantitative risk assessment (Thomas et al. 2011). Briefly, a transcriptomic POD (tPOD) represents the lowest dose at which there is a detectable concerted change in gene expression activity in response to a chemical exposure (Johnson et al. 2022a). At exposure levels below this hypothetical tPOD, the likelihood of experiencing adverse toxicological effects is minimal. The tPOD is thought to be highly protective of all potential adverse toxicological effects that can be induced by a chemical stressor, making it a potentially valuable tool in risk assessment. One of the most attractive advantages of tPODs is that they can be generated after short exposure periods yet appear to provide quantitatively comparable results to long-term tests that evaluate traditional apical endpoints, such as changes in organ weight or the formation of tumors (Thomas et al. 2012; Bhat et al. 2013; Farmahin et al. 2017; Pagé-Larivière et al. 2019; Gwinn et al. 2020; Johnson et al. 2020). Therefore, the use of tPODs in deriving reference doses could provide a pragmatic approach to increase the efficiency of toxicological evaluations.

Johnson et al. (2022a) proposed a framework consisting of 4 central principles for using tPODs in chemical risk assessment. The first principle establishes that transcriptomics can reliably detect alterations in gene expression that result from endogenous or exogenous influences on an organism. The second principle states that alterations in gene expression are indicators of adverse or adaptive biological responses to stressors in an organism. The third principle contends that transcriptomics can be employed to establish a tPOD from short-term, in vivo studies to identify a dose level below which a concerted molecular change is not expected. The fourth principle is that the use of such a tPOD will be protective in the context of chemical risk assessment.

Recently, the US Environmental Protection Agency (US EPA) Office of Research and Development introduced the EPA Transcriptomics Assessment Product (ETAP), an innovative tool aimed at generating tPODs from short-term in vivo studies. ETAP facilitates the establishment of reference values for chemicals with limited data, marking a significant milestone in integrating transcriptomic data into chemical risk assessment (EPA 2023).

Despite the potential of this approach, establishing broader consensus across studies and regulatory use-cases for rigorous and reproducible tPOD characterization remains a challenge. Some of these challenges include the need for robust study designs, appropriate analytical methods, and well-curated reference databases. Notably, there are significant variations in the bioinformatics workflows that have been used for deriving tPODs in the published literature. A more standardized approach would improve the reproducibility and reliability of the results and facilitate cross-study comparisons.

This work is from the Health and Environmental Sciences Institute (HESI)’s Molecular Point of Departure Working Group, which comprised toxicologists and bioinformaticians in the government, academic, nonprofit, and commercial sectors who are collectively involved in advancing the use of molecular PODs for regulatory applications. Here, we review the current state of the science regarding the bioinformatics workflows associated with tPOD derivation and identify sources of variation in bioinformatic approaches, uncertainty, and data gaps. We also provide recommendations for specific research to address areas of need.

## Overview of tPOD data analysis workflows

**Fig. 1.**
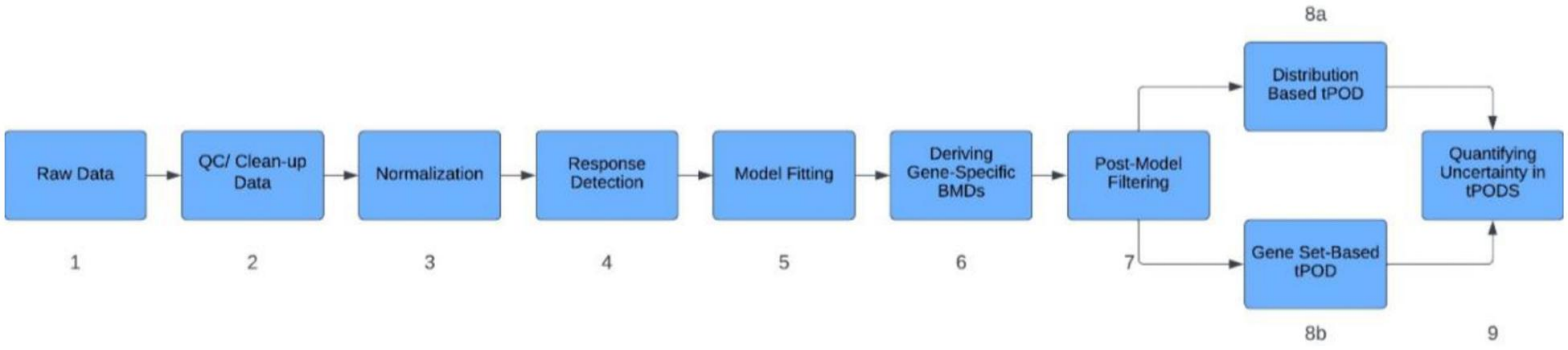
Overview of the bioinformatics workflow for determining a tPOD.

The most commonly used tPOD derivation workflow can be generalized into a 9-step process (Fig. 1) (Thomas et al. 2007; Yang et al. 2007; NTP 2018; Phillips et al. 2019). Step 1 is the input of raw gene expression data into the workflow. The term “gene” is generally used, because these data may describe different specific endpoints depending on the platform, such as mapped reads for RNA sequencing (RNA-seq), or hybridization probe fluorescence intensity for microarrays. Types of raw data also vary depending on the platform. For microarrays, raw data are usually represented in CEL files, whereas for RNA-seq, raw data are represented in FASTQ files. In Step 2, quality control (QC) and clean-up processes are applied to identify low-quality samples and filter out genes with low-expression levels across samples. This step is crucial to ensure the accuracy of the downstream analysis and may vary depending on the sequencing platform. Next Step 3, normalization ensures that the scale and distribution of the expression data are standardized across the dataset, and/or across treatment groups.

Step 4 is the response detection step and identifies genes with dose-dependent behavior and a magnitude of change above a defined threshold. Here, different statistical tests might be applied, such as ANOVA and/or Williams Trend test, in combination with filters to remove genes that do not meet minimum fold-change and *P*-value cutoffs. By removing nonresponsive genes, Step 4 reduces noise and improves the computational efficiency of the workflow. In Step 5, a BMD analysis is applied to each of the remaining genes, which fits dose–response models to the data and generates a list of gene-specific BMD values Step 6. The fitted models are then evaluated and filtered to remove any low-quality models Step 7.

The resulting list of genes with high-quality models and their associated BMDs are then used to determine transcriptome-wide tPODs. Typically, several different tPODs are calculated. These can be categorized into 2 main types: Distribution-based tPODs Step 8a or gene set-based tPODs Step 8b: See Table 1 for details. Step 9 is understanding, and if possible, quantifying the uncertainty of the tPOD.

**Table 1.** Summary of the 2 main types of transcriptome-wide tPODs.

Approach	Description
**Distribution-based tPODs**	Determined based on shape and spread of gene-specific BMDs across dose-range Examples: 5th or 10th percentile BMD, 25th gene of the distribution, first mode of the BMD distribution.
**Gene set-based tPODs**	Genes and BMDs mapped to gene sets (ontologies, pathways, coexpression networks) User-defined thresholds identify enriched gene sets tPOD based on gene set with lowest median BMD among mapped genes

Several software tools to calculate tPODs are publicly available. One of the earliest and most commonly used tools is BMDExpress, which was first described in 2007 (Yang et al. 2007). Additional tools have since been developed that have similar workflows, such as FastBMD (available in ExpressAnalyst) (Ewald et al. 2021), the Bayesian Benchmark Dose Modeling System (BBMD) (Shao and Shapiro Andrew 2018), ToxicR (Wheeler et al. 2023), BMDx (Serra et al. 2020), and DRomics (Larras et al. 2018).

Although many variations of the workflow described above are possible, there is considerable evidence that the overall approach generally produces comparable results: A strong correlation between a short-term tPOD and a traditionally derived apical chronic POD (Webster et al. 2015; Farmahin et al. 2017; Pagé-Larivière et al. 2019). These demonstrate that the overall workflow to derive a tPOD is generally stable, with most changes to the workflow having minimal effect on the relationship between transcriptomic and apical PODs. However, they also highlight several key issues that remain to be resolved.

## Outstanding data analysis data gaps and challenges

At each step along the workflow (Fig. 1), there are considerations for deriving a tPOD. In this section of the paper, we describe some of the most common approaches, gaps in knowledge, uncertainties and sources of variation, challenges, and opportunities for further research.

## Study design and dataset

For tPOD experiments, several study design factors must be considered. These designs differ from traditional toxicology tests focusing on apical endpoint PODs or those assessing specific tissue endpoints. Key technical and biological factors are essential for generating effective toxicity tests. However, the optimal design is not fixed and varies depending on the type of toxicity (e.g. general vs reproductive) and available resources.

### Technical considerations

Planning a tPOD derivation experiment begins with selecting a suitable range of dose groups, similar to BMD modeling using traditional apical endpoints (Slob et al. 2005; EPA 2012; Ewald et al. 2022). A graded response across dose levels is essential for fitting dose–response curves and estimating the BMD with confidence limits (Haber et al. 2018). A dose-range finding study may help select appropriate dose levels, ensuring at least one dose elicits a robust transcriptomic response and another shows little to no response to avoid extrapolation errors. Although a minimum of 3 treated dose groups plus controls may suffice (Kavlock et al. 1996; Slob et al. 2005; Kuljus et al. 2006; Oberg 2010; Slob and Setzer 2014; Holland-Letz and Kopp-Schneider 2015; Ewald et al. 2022; Stainforth et al. 2022), more doses may be needed for adequate transcriptomic dose–response capture.

When selecting the high dose level, it is advised to optimize the transcriptomic response while avoiding animal death and suffering. Strategies from traditional guideline toxicity studies may be sufficient; e.g. the Division of Translational Toxicology (DTT; formerly NTP) recommends a maximum tolerable dose producing less than a 20% decrease in body weight gain over the exposure period (NTP 2018). Others use a 5% to 20% effective dose as the top dose. An initial range-finding study may be necessary for appropriate dose selection. Once the high dose level is selected, scaling lower doses by log or half-log values is typical, improving tPOD estimate precision if all recommendations are met (Ewald et al. 2022).

Research has shown that top dose/concentration may impact the extent of transcriptional change (i.e. the number genes with BMDs) (Reardon et al. 2023). The authors noted this has a particular impact on tPOD methods based on percentiles (e.g. the fifth), where an increase in the number of genes with BMDs potentially shifts the tPOD higher.

Another important factor is ensuring an adequate number of biological replicates per dose group. The sample size directly influences the accuracy and precision of BMD estimates and the reliability of dose–response model fitting. Although resource constraints might necessitate using fewer replicates (e.g. 3 per dose group), the goal is to strike a balance between the number of replicates and the number of dose levels. This approach allows for a broader range of doses while maintaining sufficient replication to produce reliable model outputs, rather than sacrificing replicates entirely in favor of more dose groups (Ewald et al. 2022).

When determining the appropriate low dose for chemical testing, especially when there is no prior knowledge of the chemical’s toxicological properties or potency, it is crucial to select the lowest nonzero dose carefully. A review of the Gwinn et al. (2020) data suggests that in most cases using 8 dose levels with half-log spacing starting with a 5-d maximum tolerated dose is adequate to confidently capture a gene set POD that occurs within the dose range of the study. The EPA’s ETAP protocol follows a similar approach to Gwinn et al. (2020), adjusting the number of dose levels if prior information about the chemical’s potency exists, and ensuring a 10-fold difference between the lowest dose and the next higher one to identify a no-transcriptional-effect level. Certain chemicals, particularly pharmacological agents with a widespread in potency between their pharmacological and toxicological effects, may require an extension of the dose range beyond what was used in Gwinn et al. (2020) to fully characterize a POD. An example of such a chemical is ethinyl estradiol which has approximately 6 orders of magnitude between a 5-d maximum tolerated dose and the no bioactivity level. These rare situations can sometimes be predicted using nonanimal data or computational models like QSAR to better estimate the chemical’s potency.

General principles of sound experimental design, such as proper use of vehicles and controlling for confounders, are essential in transcriptomic experiments as in conventional studies. Technical sources of variation, such as batch effects, can increase uncertainty and biases in BMD estimation, impacting tPODs. Batch effects can arise from various factors, such as RNA extraction batches, plating effects, differences in sample preparation dates, or other types of sample grouping, leading to measurement differences between groups (Leek et al. 2010).

### Biological considerations

As in any in vivo study involving chemical exposure, it is important to consider the duration of exposure, the life stage and sex of the animal during exposure and at the time of endpoint analysis, and the selection (and processing and storage) of tissue(s). Within the context of this manuscript, the primary focus will be on rodent models, but other animal models (e.g. zebrafish embryos) and in vitro models have been used for transcriptomics. Indeed, there is an increasing focus in the ecotoxicology space using fish, amphibians, and birds (Crump et al. 2023). The complex nature of gene expression requires careful consideration of the duration of exposure. To date, previous work has suggested that transcriptional BMDs from exposures of 5 days or longer are correlated with cancer and subchronic or chronic noncancer apical BMDs, and that transcriptome BMDs stabilize in vivo within a few days of repeated dose exposure (Hester et al. 2005; Bhat et al. 2013; Thomas et al. 2013; Gwinn et al. 2020; Johnson et al. 2020).

As is the case for traditional apical studies, the exposure window reflecting the life stage of the model is another important consideration to produce meaningful transcriptomic data as the effects from exposure may manifest differently during different life stages. In general, the life stage selected for exposure should reflect the critical window for the response of concern. For example, exposure of adults is appropriate to derive a chronic toxicity or carcinogenicity POD but may not be relevant for a developmental toxicity POD. The sex of the animals used should be considered, as chemicals can exhibit sex-dependent effects. A comprehensive analysis in both males and females is necessary to capture biological responses in sex-specific tissues such as reproductive organs (e.g. gonads, uterus) or other organs like kidneys which may have sex-specific differences.

The selection of appropriate tissues is essential to ensure accurate and relevant information is obtained within a transcriptomic study. In rodents, the liver and kidney have been previously considered as potential “sentinel” tissues that, after a 5-d exposure, produce tPODs that reflect apical BMDs (Gwinn et al. 2020); however, the validity of using an organ as a proxy for another organ requires additional research. When mechanism or mode of action is unknown, it is recommended that a broad scope of organs is selected for transcriptome analysis to capture the breadth of potential toxicological effects.

## Platform

Multiple technologies exist for transcriptomic profiling, including microarrays and RNA-seq. Steady increases in the throughput and cost efficiency of RNA-seq have resulted in its rise in popularity. However, there are a large amount of public and private data derived from microarrays. There are also qPCR-based platforms available (e.g. Qiagen’s RT2 Profiler Array) to use for targeted panels of genes. More recently, targeted RNA-seq methods (e.g. TempO-seq, Yeakley et al. 2017; RASL-seq, Bhuller et al. 2021) have been developed for an increasing number of mammalian and ecologically relevant species, wherein predetermined probe sequences are hybridized to sample RNA, and subsequently converted to a sequencing library.

Transcriptomic platforms differ in a variety of ways, including signal-to-noise, dynamic range, and number of transcripts captured, each of which may impact the optimal analysis choices for dose–response modeling (Black et al. 2014; Zhao et al. 2014; Nault et al. 2015; Webster et al. 2015). Each platform has different detection limits, accuracy, background levels, and biases for measuring the expression of transcripts. Therefore, downstream data processing methods will likely need to be reassessed and optimized to each platform, and across a variety of experimental design paradigms. The preprocessing steps used to generate expression data, such as background correction and probe filtering for microarrays or alignment and quantification for RNA-seq, can reduce variation but may introduce uncertainty due to the choice of normalization method used and from other user-defined cut-offs (Mezencev and Auerbach 2020). Platform-specific normalization methods may also reduce technical variation. Several important considerations for tailoring dose–response modeling methods to specific transcriptomic platforms are highlighted below.

One critical choice for all sequencing-based methods is sequencing depth (i.e. the number of reads sequenced per sample). Typically, increasing the number of samples multiplexed into a single sequencing lane increases throughput, but decreases sequencing depth, which in turn leads to reduced accuracy of gene expression estimation (Su et al. 2014; Everett et al. 2022). This is particularly true for transcripts at the lower end of the expression range. Previous efforts have established guidelines for the minimum sequencing depth for general-purpose application of RNA-seq (Su et al. 2014), but there remains a need to evaluate how sequencing depth impacts the accuracy of read-outs in dose–response modeling, particularly for overall tPOD determination. RNA-seq also includes several other methodological choices, such as read length and whether to sequence one or both ends of each RNA fragment, which have major impacts on the feasibility and accuracy of deconvoluting transcripts resulting from different splicing events within a gene.

Apart from sequencing depth, both platform and experimental design affect signal-to-noise variations in genes. Microarrays have a narrower dynamic range than sequencing, which may reduce the estimation of effect sizes across the transcriptome (Rao et al. 2018). Probe design in microarrays and targeted RNA-seq also affects expression accuracy for some genes. Additionally, the genomic platform used can impact the choice of statistical model and assumptions made throughout the analysis, leading to different results. Technology-specific biases can also be present, such as targeted probe sets vs whole transcriptome.

Genome coverage also varies across different transcriptomic platforms, which may impact the ability to detect a broad array of biological responses. Targeted RNA-seq methods such as TempO-seq allow the use of reduced representation probe sets, such as the S1500+ sentinel gene sets (Mav et al. 2018; Balik-Meisner et al. 2019), which encompasses ∼3,000 genes chosen to maximize coverage of the majority of known biological pathways. As with number of samples, there is an inherent trade-off between sequencing depth and number of transcripts profiled using targeted RNA-seq technology. Sentinel gene sets have the advantage of decreasing study cost and increasing throughput, and previous work has shown that these platforms can recapitulate the major findings (e.g. tPOD and pathway analyses) of whole transcriptome platforms (Bushel et al. 2018; Lee et al. 2021). Previous work also established that the sequencing depth of the rat S1500+ platform can be reduced to as low as half a million reads per sample while maintaining highly correlated fold-change values with standard RNA-seq approaches (Everett et al. 2022). Studies suggest that a well-selected sentinel gene set will result in quantitatively similar tPODs as the whole transcriptome, especially when distribution-based methods are employed (Costa et al. 2024). However, the reliability of pathway results when using a reduced transcriptome remains unclear.

We also note that the use of nonmammalian organisms (e.g. species used for ecological toxicity studies) presents unique challenges. Some species tend to have less mature pathway annotations across the majority of known genes, and therefore the use of pathway-based tPODs (discussed below) may not be an effective option for these species. Although ortholog mapping is an effective tool for applying pathway knowledge across vertebrate genomes, some of the species used for ecological toxicity testing (e.g. algae and invertebrates) have metabolic and regulatory systems that are not conserved in vertebrate species (Seebacher 2018; Hakeemi et al. 2022); therefore, filling in the gaps of gene annotation for ecological species is not as simple as identifying nearest orthologs in species with more extensive genome annotations.

A critical question is ultimately whether the choice of platform has a major impact on the tPOD. Although there has been extensive work demonstrating strong concordance between transcriptomic platforms in general applications, there are limited case studies to date addressing this question. Thus far, the results suggest that tPOD determinations are highly reproducible within an order of magnitude when compared across platforms (SEQC/MAQC-III 2014; Bushel et al. 2018). Work examining the dose-dependent transcriptional response to furan found that RNA-seq and microarrays produced highly concordant overall tPOD values (within 2-fold) although the specific genes and pathways involved in the tPOD calculation varied considerably (Black et al. 2014; Webster et al. 2015). Similar concordance has even been demonstrated across species for myclobutanil, where a tPOD estimated in rat liver using RNA-seq was within 4-fold of the tPOD previously estimated in mouse liver using microarrays (Bhat et al. 2013; LaRocca et al. 2020). An evaluation of existing studies found that the concordance between tPODs and apical PODs was consistent across different transcriptomic platforms (Chang et al. 2024). Although these studies suggest that overall tPOD values have cross-platform consistency, further research is merited to explore whether the choice of platform may have an impact on mechanistic inference and biological interpretation specifically in the context of dose–response experiments.

## Data preprocessing

We define preprocessing as the steps that occur after platform-specific data extraction (e.g. feature extraction from microarray or sequence alignment for RNA-seq) and before dose–response modeling. The purpose of preprocessing is to prepare the gene expression data for downstream analyses by applying a series of data cleaning and QC steps. The 3 main objectives of preprocessing are: (i) identify and remove poor-quality/outlier samples; (ii) normalization; and (iii) filter low-detection and/or low-variance genes from the analysis.

Prior to processing the data, it is important to consider their distribution. Gene expression data can have various distributions depending on the platform used or processing steps applied. In many (but not all) cases, transcriptomics data will have log-normal distributions. However, many of the downstream statistical procedures described below assume a normal distribution. Therefore, transcriptomics data are often log-transformed to make the data more symmetric with a normal variance distribution. It is important to be aware of the distribution of the data throughout the various processing steps (since transformations are often included in some of these procedures like normalization) and to ensure that data meet the assumptions of any statistical procedure being applied.

In preprocessing, the first step is to eliminate outliers from the dataset. These outliers, caused by technical errors and other uncontrolled experimental variables, can significantly impact analysis quality. Removing them is crucial to minimize noise, enhance signal detection, and guarantee consistent, reliable results. Assessment methods for sample quality vary, being either platform-dependent or independent. For instance, microarray analysis involves checking background fluorescence, signal-to-noise ratios, control feature performance, and probe saturation. In contrast, RNA-seq analysis focuses on read quality, distribution, and alignment rates. The objective is to identify and discard samples that fail to meet established quality standards.

Platform-independent methods for identifying low-quality or biological outliers usually involve inter-sample comparisons and can include determining sample-to-sample correlation coefficients or multivariate clustering analysis such as principal component analysis or hierarchical clustering. Thresholds for the identification of outliers should be determined prior to analysis. Harrill et al. (2021a) developed several QC criteria specifically for identifying and removing low-quality samples from RNA-seq studies. These included criteria for read mapping rates and read count saturation and distribution. The omics data analysis framework for regulatory application (R-ODAF) also has a set of quality assurance and QC parameters for omics data specifically developed for regulatory use cases (Verheijen et al. 2022).

## Normalization

Normalization is performed to reduce nonbiological technical biases and variations that can arise during the experiment and data generation processes. Normalization ensures that the data accurately reflects the true biological signal and enables valid comparisons between samples and across dose groups. Selection of a normalization method is often dependent on several factors including the platform and experimental design. Some common methods for normalizing microarray data include Loess (Smyth and Speed 2003) and robust multi-array average normalization (Irizarry et al. 2003). Transcripts per million or reads per kilobase million are popular methods to normalize RNA-seq data (Corchete et al. 2020; Zhao et al. 2021). Both methods can normalize based on library size and gene length.

Studies have investigated the effects of normalization methods on transcriptomic dose–response analysis outputs. For example, Pagé-Larivière et al. (2019) conducted a dose–response analysis of microarray data from fish exposed to estrogenic compounds and found that the resulting tPODs were minimally affected across 4 normalization methods. However, the specific list of differentially expressed genes (DEGs) that were identified as dose-responsive varied significantly. Similar effects on DEG lists were also reported by Guo et al. (2006) for a standard DEG analysis. Overall, the selection of normalization method is unlikely to have a strong influence on the distribution of gene-BMDs, and therefore on tPOD values. However, due to the instability of the DEG lists, normalization may have a greater influence on any mechanistic interpretation of the results. Additional research will be required in cases where mechanistic interpretation is performed to determine approaches to DEG-list determination that are more robust to variations in normalization.

## Filtering genes with low detection and low variance

The filtering preprocessing step involves excluding genes with low-detection levels or low variance to remove noise and irrelevant data. This process simplifies data complexity, conserves computational resources, and enhances efficiency. Genes poorly or inconsistently detected often exhibit high variance across samples and are therefore difficult to fit any dose–response model with sufficient confidence. Additionally, genes can be filtered by their detection rates, which is the proportion of samples where they are detected. Filters are sometimes applied to remove genes not expressed in every sample. Genes with low variability across samples tend to carry little meaningful information because they are nearly constant across treatment groups. Low-variance filters in particular can improve the sensitivity and reduce the false discovery rate for DEG analysis (Bourgon et al. 2010). A data-driven approach to gene count filters for RNA-seq data has been proposed to increase detection power (Rau et al. 2013). Overall, careful consideration must be made when selecting filter threshold values.

We are unaware of specific studies that have measured the effect of different low-detection or low-variance filters on transcriptomic dose–response modeling outputs. As long as overly stringent filters are not applied, these filters are expected to have a beneficial impact, although empirical studies could be conducted to identify optimal settings.

## Response detection filter

For certain programs like BMDExpress, the next step after preprocessing is the application of a response detection filter. Its purpose is to remove genes that do not change across treatment groups to separate true signal from noise and retain genes that exhibit dose-responsive behavior. This step reduces noise, limiting the total number of genes to model, and reducing computational demands in downstream steps.

Historically, the 2 main statistical response detection filters in transcriptomic research have been the standard ANOVA and the Williams Trend test. ANOVA checks if gene expression significantly differs from control in any dose group, whereas the Williams Trend test looks for increasing or decreasing expression trends with dose escalation. Both methods require a *P*-value cutoff, potentially with multiple test correction and/or a fold-change cutoff. The NTP’s current recommendation is to use a Williams Trend Test combined with an effect size filter, with thresholds tailored to each transcriptomic platform (NTP 2018). Commonly, a William’s Trend Test with *P* < 0.05 (10,000 permutations) and a fold-change >1.5 is used (NTP 2018; Chang et al. 2024).

Studies like those by Webster et al. (2015) and Farmahin et al. (2017) have explored multiple filtering and thresholding approaches. Webster et al. found that increasing filter stringency marginally reduced mean BMDs and benchmark dose lower confidence limit (BMDLs) in gene expression studies, without significantly affecting tPOD values. Farmahin et al. observed that FDR-corrected *P*-values provided better tPOD predictions than uncorrected ANOVA. However, these studies predate the adoption of newer filters like the Williams Trend test, now commonly used for selecting genes for BMD modeling.

The latest version of BMDExpress software (v3.0) includes a new method for statistical filtering of features called the “Curve Fit Pre-Filter.” This method uses the ToxicR curve fitting process to fit models to all features with a SD-based benchmark response (BMR) set to a high threshold, such as 2 SDs (Wheeler et al. 2020). Although typically combined with a fold-change threshold, this combination is not mandatory. Features with the best-fit model BMDs below the highest dose pass the filter and proceed to the modeling step. This approach leverages all the data and constrains the response shapes to biologically plausible ones, making it potentially more stringent than the Williams Trend test with a fold-change threshold, provided the Curve Fit BMR thresholds are set appropriately. However, there are no published comparisons of data from matching samples using this method.

It is generally accepted that more stringent response detection filtering leads to less modeled noise and more accurate predictions. However, a balance is necessary to avoid removing truly responsive features. Inappropriately stringent filtering can introduce significant variation in the number of genes that pass to the modeling step, causing uncertainty and variance in the final tPOD (Ewald et al. 2022). Standardization of response detection filters, as demonstrated in the ETAP scientific support document, can help address this issue (Chang et al. 2024). ETAP’s workflow optimization aimed to maximize concordance between tPODs and chronic apical PODs, but different optimization criteria may be suitable for other cases.

### Gene-specific model fitting and BMD derivation

After upstream processing and sufficient filtering (e.g. statistical and effect size), the data are subjected to model fitting using regression-based approaches (Thomas et al. 2007). When fitting the data, constant variance is assumed as the data are typically log-transformed. If data are not log-transformed, then there should be consideration of nonconstant variance. The most common models employed are parametric (i.e. predefined mathematical form) models for continuous data that represent plausible biological dose–response shapes. The models typically employed are the linear (polynomial 1), Hill, power, and exponential. Often these models can be used with limited impact on the results due to adaptability of the fits (Thomas et al. 2011; Bianchi et al. 2021). Historically, the fitting process has used maximum likelihood estimation to identify the best fit for each model. BMD values for each fitted model are estimated based on a predefined threshold of response, or BMR.

The BMR is a predetermined change in the measured endpoint (i.e. gene expression) used to determine the BMD. For traditional apical endpoints, a BMR deemed “biologically significant” is typically selected based on expert evaluation. However, the biologically relevant change for every gene in the transcriptome remains unknown, leading to a generalized approach. The US EPA recommends setting the BMR to 1 SD above the modeled control level when the direction of the adverse effect is known (EPA 2012). For gene expression levels where either direction of change could be potentially adverse, the ETAP standard methods recommend 1.349 SDs, approximating a 10% increase in the risk of “abnormal” values (Thomas et al. 2007; Jensen et al. 2019). Critics argue that SD-based BMR selection is susceptible to experiment-specific and inter-individual variation, resulting in inconsistency. Alternative approaches, such as relative deviation and effect size theory, propose considering endpoint variance across multiple studies for more reproducible BMD values (Slob 2017; Zeller et al. 2017). Further work is needed to evaluate if the use of relative deviation-based BMR selection yields greater interstudy consistency in the context of genomic dose-response.

After fitting models to data and selecting a BMR for each gene, gene-specific BMD, BMDL, and benchmark dose upper confidence limit (BMDU) values are determined. Models that produce nonzero and finite estimates of the BMD, BMDL, and BMDU are typically subject to model selection by determining the “best model.” Earlier workflows, which are embodied in the BMDExpress software, recommended a combined nested likelihood ratio test for the polynomial models followed by comparison to the rest of the fitted models (Thomas et al. 2007), where the final best model was selected based on the lowest Akaike Information Criteria (AIC) score. More recently, all models with convergent values are compared and selected based on the lowest AIC. The best-fit model is then used to report the gene-specific BMD, BMDL, and BMDU. Models can also be evaluated based on a global goodness of fit *P*-value, where *P* > 0.1 is indicative of an adequate fit. An additional criterion identifies instances where the BMDL and BMDU are unreasonably wide (e.g. BMD/BMDL >20 or BMDU/BMDL >40), which is indicative of extreme model extrapolations due to suboptimal fits. In cases where the Hill model is identified as the best fit, there is an additional flag based on the parameter *k*, which describes the dose corresponding to the median increase in the dose-response. If this value is less than 1/3 of the lowest nonzero dose, then the Hill model is rejected and the next best-fit model is selected assuming a minimal quality of fit. The updated fitting procedures in BMDExpress 3 and ToxicR employ improved optimizers, which reduce the occurrence of Hill model fits with extremely low BMD estimates (Wheeler et al. 2023). The best-fit approach is currently employed in several dose-modeling programs including BMDExpress, BMDx, and FastBMD (available in ExpressAnalyst). Model uncertainty is also an important consideration, as different models may fit the data equally well but result in different BMDs (West et al. 2012; Ringblom et al. 2014; Wheeler et al. 2020; Ji and Shao 2022; Ji et al. 2022). Moreover, postfiltering, such as filtering features based on the BMD to BMDL ratio or model fit *P*-value, may introduce bias into the resulting tPOD estimate by shifting the tPOD distribution (Webster et al. 2015).

Despite being the historical standard, the “best model” approach has its challenges. Picking a single “best model” can result in bias and underestimates the size of the confidence intervals for the BMD estimate as outlined in West et al. (2012). For these reasons, several new approaches for model fitting and potency estimation have been developed. Model averaging, an alternative approach seeing widespread acceptance in dose–response modeling for other types of endpoint data, averages information from all parametric model fits (Hoeting et al. 1999; Jensen et al. 2019; Ji et al. 2022; Wheeler et al. 2022). Model averaging is now included in software packages like BMDExpress (v3.0), BBMD, Proast, and ToxicR.

Model averaging applies to a finite set of parametric models, but nonparametric models like the Bayesian nonparametric ALOHA approach offer advantages in nonmonotonic dose responses (Davidson et al. 2022). ALOHA (aggregated local extrema splines for high-throughput dose–response analysis), developed for tPOD derivation, flexibly describes dose–response relationships while retaining high-dose cytotoxicity data. However, these models lack a global goodness of fit *P*-value, using the coefficient of determination (R2) instead, with >0.6 as a preliminary quality threshold. More research is needed before fully integrating such methods into transcriptomic dose–response modeling pipelines.

## Derivation of transcriptome-wide POD

Whereas traditional studies on apical effects typically evaluate the dose-response of a relatively small number of endpoints, transcriptomic dose–response studies assess thousands of genes simultaneously. To address this, the collection of gene-specific BMDs that results from the model fitting step are parsed down into a single transcriptome-wide POD (i.e. a tPOD). In general, the most common approaches for aggregating gene-specific BMDs to derive a tPOD fall into 2 categories: Distribution-based methods and gene set-based methods.

Distribution methods for deriving a tPOD are based on evaluating the spread of BMDs from every dose-responsive gene simultaneously. Generally, these approaches aim to identify a point (or a range, when considering confidence intervals) along the dose-response where a coordinated alteration in the entire transcriptome is first initiated, without any specific mechanistic interpretation. The simplest distribution methods define the tPOD based on the rank order of the BMDs, such as the 25th ranked BMD or 5th percentile BMD (Reardon et al. 2023). Other methods include the “lowest consistent response dose” (Crizer et al. 2021), “maximum-curvature” (Johnson et al. 2022b), and “first-mode” approaches (Pagé-Larivière et al. 2019). These methods evaluate the cumulative frequency of BMDs across the dose-response to identify an inflection point in the number of responsive genes. Because these methods are distribution-based, they are relatively simple, generally have low variance, and can be computed even when there is a relatively weak gene expression response (Pagé-Larivière et al. 2019; Reardon et al. 2023). Additionally, knowledge of biological gene function is not required for these approaches, they can be applied in species with poorly annotated genomes.

A challenge related to distribution-based approaches stems from their sensitivity, which allows them to be computed for even very subtle responses. This heightened sensitivity has raised concerns about the need to establish a minimum threshold of dose-responsive genes before employing distribution-based methods (Villeneuve et al. 2023). Ramaiahgari et al. (2019) proposed a minimum number of dose-responsive genes based on the SD observed in a selection of “non-toxic” chemicals. This cutoff is likely to be platform and model specific and is also highly dependent on the accurate selection of “non-toxic” test substances. An alternative approach uses semiparametric Bayesian models (Reynolds et al. 2020). The increased level of robustness of this model was reasoned to permit the reliable identification of a tPOD without the need to predefine a minimum number of dose-responsive genes. The calculated tPODs demonstrated a strong correlation with nontranscriptomic PODs in an in vitro model. Overall, further exploration examining concordance using in vivo models is warranted for this approach.

Gene set-based methods for tPOD determination involve grouping dose-responsive genes into pathways or gene sets (e.g. Gene Ontology, REACTOME, BioPlanet, KEGG), based on factors like biochemical pathways, molecular signaling networks, or transcription factor regulation. The underlying idea is that genes involved in the same process or pathway tend to be coregulated. Thus, it is assumed that dose-responsive behavior in multiple genes from the same gene set is evidence that the pathway/process has been perturbed, that the dose marking alteration of the most sensitive pathways/processes is a conservative estimate of where a system-wide concerted molecular change in the transcriptome begins, and that the gene sets that are altered at the lowest doses are the most directly related to a substances mechanism of action or molecular initiating event. Generally, the steps involved in these methods include: (i) Mapping genes to gene sets; (ii) Identifying responsive sets; (iii) Summarizing gene BMD values within sets, usually by median BMD value of the gene set; and (iv) Selecting the lowest summary value as the tPOD (e.g. the lowest median BMD). In some cases, this approach has the potential to provide mechanistic insights into transcriptomic dose-responses, identifying sensitive pathways to infer a chemical’s action. Although there have been examples where a connection between the most sensitive gene set tPODs from a short-term transcriptomic study could be directly linked to apical endpoints of concern, the majority of studies have not attempted to infer such associations (NTP 2018). Nevertheless, the strong quantitative correlation between the value of gene set-based tPODs and traditional apical PODs has been repeatedly demonstrated (Chang et al. 2024).

The most commonly applied gene set-based approach is that recommended by the NTP in 2018 (NTP 2018). Specifically, this approach defines the tPOD as the lowest median BMD among gene sets with a minimum of 3 dose-responsive genes that affect at least 5% of the gene set. The EPA adopted a similar approach in 2024 (Brennan et al. 2024), anchoring the tPOD to the gene set with the lowest median BMD and at least 3 dose-responsive genes, but ultimately using the more protective median BMDL as the overall tPOD. Generally, these approaches yield tPODs that are highly correlated to distribution-based tPODs (Farmahin et al. 2017; Pagé-Larivière et al. 2019; Reardon et al. 2023; Costa et al. 2024).

For extracting mechanistic information, it is unclear whether simple gene set enrichment based on broadly defined functional classifications (e.g. gene ontology terms, especially at higher levels of the GO hierarchy) is an effective approach for tPOD studies because this approach does not consider the dose and temporal relationships between genes. Costa et al. (2024) showed that randomized gene sets based upon the structure of biological knowledge-derived gene sets produced similar tPODs when compared with the original biological knowledge-derived gene set counterparts, when the NTP gene set enrichment criteria are used (i.e. ≥3 genes and ≥5% coverage). They suggest that little biological information is used in the gene set-based tPOD generation approach, unless more stringent enrichment criteria are used.

Methods interrogating coexpression networks (Sutherland et al. 2018; Ewald et al. 2020) appear to have benefits over other types of gene set-based methods, though more work is required to determine if they can consistently produce reliable mechanistic information for transcriptomic dose–response studies (Basili et al. 2022).

Recent publications highlight alternative mechanism-based approaches, like Harrill et al. (2021a) and Basili et al. (2022), who proposed aggregating normalized gene expression data into pathway signature scores via single-sample GSEA prior to dose–response modeling (Harrill et al. 2021a; Basili et al. 2022). This method, which derives summary BMDs from these signature scores, showed increased inter-sample correlation and better alignment with previous high-throughput in vitro assay results (e.g. ToxCast; Paul Friedman et al. 2020), compared with gene set mapping postmodeling. Basili et al. (2022) aggregated gene responses into latent variables (LVs) informed by predefined biological pathways/signatures and then modeled the response of the LVs to derived potency estimates. These responses had a moderate correlation with approaches like BMDExpress.

## Quantifying uncertainty of tPODs

Dose–response modeling of a single gene or other endpoint typically includes an estimated confidence interval around the POD that reflects the degree of uncertainty given the underlying data (Moerbeek et al. 2004). However, the most common software platforms for transcriptomic dose–response modeling lack any tools for extending this estimate of uncertainty to the overall computation of the tPOD aggregated over multiple genes, which limits insight into the robustness of this estimate for a given dataset.

In instances where the tPOD is based on a single gene or signature, the confidence intervals can be derived directly from the endpoint-specific fitted model. However, this approach only characterizes the uncertainty of the single endpoint and is unlikely to represent the response uncertainty across the entire transcriptome. Even when the tPOD comes from a specific gene or signature, the robustness of that estimate still depends on other genes with similar BMD. Calculating true confidence intervals for tPODs in this complex workflow might be difficult, though bootstrapping or heuristic approximations may be helpful options. Bootstrapping appears to be one of the more common approaches to estimating tPOD confidence intervals. It has been used for both distribution-based and gene set-based methods (Farmahin et al. 2017; Pagé-Larivière et al. 2019), though the precise methods vary depending on how the tPOD is determined. For example, in the case of distribution-based tPOD, bootstrapping is employed across the entire set of dose-responsive genes. Conversely, for gene set-based tPODs, bootstrapping is typically confined to the smaller list of genes within the gene set of interest, which may not reflect the response uncertainty across the entire genome. Subsampling approaches can also be used for studies with larger dose and replicate numbers (Ewald et al. 2022; Villeneuve et al. 2023).

The Bayesian-based methods (Reynolds et al. 2020) include 2 measures of uncertainty for each dose-responsive gene: A heuristic measure of the confidence in the existence of a dose–response relationship between a gene and the treatment, which they call the concentration dependency score, or CDS, and credible intervals describing the distribution of the computed tPOD.

In general, deriving confidence intervals on tPODs is a more recent application, but several promising approaches are already available to provide reasonable estimates of uncertainty and variation. Thus, it is recommended that approaches to measure tPOD confidence be made available in more transcriptomic dose–response analysis software.

## Discussion, conclusions, recommendations for path forward

The development of tPOD approaches represents a significant step toward integrating transcriptomics into the quantitative risk assessment paradigm. In evaluating the current landscape of transcriptomic dose–response studies, it is evident that the bioinformatics and data processing steps employed enable the derivation of tPODs that are generally well-correlated with apical PODs and can provide protective estimates of chemical exposure levels that are unlikely to cause adverse effects. Although some research questions remain, current data suggest that tPODs will be health protective, with additional studies likely to reinforce this conclusion. However, to ensure their reliability across a wider range of regulatory contexts and use cases, there is a need to review current practices and identify critical knowledge gaps and research priorities to ensure that tPODs are robust and reproducible. Here, we summarized the most common bioinformatic methods used to derive a tPOD, and highlighted the most important gaps that should be addressed in order to update best practices. Table 2 summarizes the knowledge gaps and research priorities identified in this paper across the different sections.

**Table 2.** Knowledge gaps and research questions.

Section	Knowledge gap/research priority
Study design, technical considerations	What is the ideal experimental design in terms of dose selection and group size? Is there an ideal maximum dose level? • What is the optimal number of replicates? • What is the ideal dose spacing? Can these recommendations be confirmed as optimal?
Study design, Biological considerations	Regarding exposure and sampling time: • Is there an optimal time (e.g. after a chemical-dependent steady state has been reached) to determine tPODs? • How stable/dynamic are tPODs over time? Regarding tissue selection: • Is there a minimum set of tissues that should be analyzed for in vivo studies? Regarding cell type selecting: • What are the minimum number of cell types that should be analyzed for in vitro studies?
Platform	Does platform choice affect tPOD results? • One study suggests that tPODs are consistent between microarray and RNA-seq platforms (Webster et al. 2015). • How does platform affect mechanistic interpretations?
	How does sequencing depth affect tPOD accuracy/reproducibility? Are certain tPOD methods more vulnerable to differences in sequence depth (distribution vs gene set-based analysis)? Do other methodological options (read length, strand direction, etc.) for RNA seq affect tPOD results?
	Does genome coverage affect tPOD (number and selection of genes)? Does the degree of genome annotation affect tPODs?
Data preprocessing Normalization	Normalization does not seem to affect final tPOD value. However, it does affect the DEG list. Does this have an impact on mechanistic interpretations?
Data preprocessing Prefiltering low-detection/variance genes	Does prefiltering impact the downstream false-positive/negative rate? If so, what are best practices for selecting/optimizing the prefiltering criteria?
Data preprocessing Response detection prefilter	Is there a minimum treatment-induced transcriptomic response that should be met before transcriptomic dose–response modeling can/should be applied? What is the impact of various response detection filters on assay sensitivity, tPOD stability, and mechanism interpretation?
Model fitting and BMD derivation	A cross-study evaluation of gene-specific BMRs should be conducted, and experts should work to develop consensus recommendations for BMR selection for modeling of individual genes.
	What is the impact of including of nonparametric/nonmonotonic models on tPODs?
	Does postmodel filtering affect the sensitivity/specificity of the assay, or introduce any bias to the tPOD distribution?
Derivation of transcriptome-wide POD	Investigate the need to establish a minimum threshold of dose-responsive genes before employing distribution-based methods
	Compare robustness/reproducibility of various tPOD derivation methods
	Investigate the effect of modulating pathway enrichment methods and stringency criteria (e.g. 3 gene/5% minimum) on assay sensitivity, tPOD value, and mechanistic interpretations
	Do any of the different gene sets (GO, pathways, coexpression networks) or gene set enrichment approaches (e.g. dose–response modeling before/after enrichment) provide more reliable mechanistic interpretations?
Quantifying uncertainty of tPODs	Establish recommendations/best practices for quantifying uncertainty around tPOD. Can a general approach be adopted, or will it be dependent on how tPOD is derived? Including some measure of tPOD uncertainty should be included in the common software platforms to encourage consistent reporting.
Reporting	Update the OECD omics reporting framework (Harrill et al. 2021b) to address additional components of tPOD derivation as outlined in this report.

In addition to addressing these gaps and priorities, another pragmatic step would involve updating standardized reporting frameworks. The OECD has made considerable progress creating a reporting framework for transcriptomics, which includes a data analysis reporting module for dose–response modeling studies (Harrill et al. 2021b). However, this reporting framework was largely developed based on the 2018 NTP recommendations. For example, the Data Analysis and Reporting Module for BMD Analysis was designed specifically to capture the information related to deriving BMDs for individual molecules or sets of molecules, and by design does not include fields for reporting on distribution-based tPOD methodology nor on more complex methods for uncertainty characterization beyond the standard confidence interval used. Thus, the OECD omics reporting framework should eventually be expanded to promote consistent reporting of all the bioinformatic workflow variations discussed above.

Finally, it should be noted that other high-content technologies, including metabolomics, proteomics, and imaging-based phenomics (e.g. Cell Painting) have emerged as tools for toxicology that can also be used to derive molecular PODs (Crizer et al. 2021; Nyffeler et al. 2023). Although discussion of these is beyond the scope of this paper, many of the aspects addressed above will be relevant to dose–response analysis of the data they generate, although they will have their own considerations for their respective bioinformatics pipelines.

In conclusion, the field of transcriptomic dose–response modeling and tPOD derivation is undergoing rapid evolution, driven by technological and methodological advancements. These advancements are paving the way for the development of more precise and predictive models with tangible applications in risk assessment. Initiatives like EPA’s ETAP mark a significant milestone in the integration of transcriptomic data into chemical risk assessment, with further groundbreaking applications anticipated in the coming years. Additionally, a mindset change is needed to accept these types of data and others in an integrative approach over traditional apical endpoints (Bhuller et al. 2021). Although certain challenges persist, ongoing research efforts and global collaborations hold promise for effectively addressing these obstacles. Through continued refinement and collective efforts, we anticipate the emergence of more robust and reliable bioinformatic approaches to deriving tPODs in the near future.
